# Risky sexual behavior and associated factors among out-of-school youths in Addis Ababa, Ethiopia; mixed methods study

**DOI:** 10.1186/s12978-024-01808-y

**Published:** 2024-06-05

**Authors:** Samuel Dessu Sifer, Milkiyas Solomon Getachew

**Affiliations:** 1 Department of Public Health, Yekatit 12 Hospital Medical College, Addis Ababa, Ethiopia; 2 Department of Public Health, College of Medicine and Health Sciences, University of Gondar, Gondar, Ethiopia

**Keywords:** Risky sexual behavior, Out-of-school, Youths, Associated factors, Addis Ababa

## Abstract

**Introduction:**

Sexual risky behaviors, as defined by the World Health Organization, encompass a spectrum of sexual activities that heighten the likelihood of negative outcomes related to sexual and reproductive health. Despite the implementation of various healthcare programs and interventions, youths continue to encounter challenges in accessing reproductive health services. Consequently, they remain vulnerable to engaging in high-risk sexual behaviors; 50.36% of adolescents in Ethiopia. Therefore, this study was aimed to determine the prevalence of risky sexual behavior and associated factors among out-of-school Youths in Addis Ababa, Ethiopia; 2023.

**Methods:**

A community based cross sectional mixed methods study was conducted among 701 youths in Addis Ababa from September 1st to 30th, 2023. The quantitative data were collected through face to face interview using a pre-tested structured questionnaire, while qualitative data were gathered through in depth interviews and focus group discussions. For the quantitative study, the study samples were chosen using systematic sampling. Conversely, purposive sampling was employed for the qualitative study. Variables with *P*-value *≤* 0.25 in the bivariate analysis were considered as candidates for the multivariable analysis. Statistical significance was declared at a *P*-value less than 0.05.

**Results:**

The prevalence of risky sexual behavior among out of school students in Addis Ababa was 40.6% (95%CI: 36.8, 44.1). Age 15–19 years (AOR: 2.52; 95%CI: 1.61, 3.94), being female (AOR: 2.84; 95%CI: 1.93, 4.18), fathers who were unable to read and write (AOR: 4.13; 95%CI: 2.04, 8.37), alcohol consumption (AOR: 2.07; 95%CI: 1.33, 3.19), peer pressure (AOR: 2.59; 95%CI: 1.81, 3.72), live together with either of biological parent (AOR: 2.32; 95%CI: 1.52, 3.55), watching pornography (AOR: 2.10; 95%CI: 1.11, 3.97) and parental monitoring (AOR: 0.59; 95%CI: 0.39, 0.90) were factors associated with risky sexual behavior.

**Conclusion and recommendations:**

A lower prevalence of risky sexual behavior compared to prior research efforts. Age, gender, educational level of the husband, alcohol consumption, peer pressure, living arrangements, exposure to pornography, and family monitoring emerged as significant factors associated with risky sexual behavior. Therefore, government should prioritize strategies to reduce substance use, mitigate the impact of watching pornography, and enhance parent-youth connectedness.

## Introduction

According to the World Health Organization (WHO), youth encompasses individuals within the age group of 15 to 24 years [1]. There are 1.2 billion young people aged 15 to 24 years, accounting for 16% of the global population. Africa represents 19% of the worldwide youth population [2]. Investing in the health of youth is not only enhances community well-being but also contributes to a nation’s stability, development, and prosperity [3]. Given their relative lack of experience, limited reproductive health knowledge, and discomfort in accessing reproductive health services or discussing related matters with partners, youths face unique challenges compared to adults [4]. As they transition to adulthood, filled with aspirations and building their future social and academic paths, neglecting their social and reproductive health places them at risk of engaging in high-risk sexual practices [5].

High-risk sexual practice (HRSP) encompasses behaviors that heighten an individual’s vulnerability to sexually transmitted infections, psychological issues, and unintended pregnancy with its associated consequences [6]. The disproportionate burden of new HIV infections among youths globally, particularly in sub-Saharan African countries where the figure rises to a staggering 80%, underscores the urgent need for targeted interventions and comprehensive education strategies [1]. Factors contributing to this alarming statistic include inadequate access to sexual health services, limited awareness about HIV prevention methods, cultural and societal norms that stigmatize discussions about sex and HIV, as well as economic disparities that hinder access to healthcare and education [1, 6–8]. Addressing this issue requires a multifaceted approach, including scaling up HIV prevention programs tailored to the needs of young people, promoting condom use and access to HIV testing and treatment, empowering youth through education and economic opportunities, and challenging social norms that perpetuate stigma and discrimination against those living with HIV [8].

In Ethiopia, comprehensive sex education, youth friendly services, community outreach and awareness campaigns, integration of HIV services, Empowerment programs and policy and legal reforms were implemented to minimize risky sexual behavior. Despite these implementation programs, the prevalence of risky sexual behavior was more than 50% and various factors have influenced the sexual behavior of youths, and it is crucial to comprehend these predisposing factors for the formulation of more effective intervention strategies and policies [9]. Nevertheless, in Ethiopia, cultural, historical, legal, and religious prohibitions have rendered discussions about sex taboo. Consequently, researchers studying sexual issues face numerous challenges, resulting in a limited number of studies in this area in Ethiopia. Hence, the primary aim of this study is to determine the prevalence of and predisposing factors contributing risky sexual behavior and associated factors among out-of-school youths (OSY) in Addis Ababa.

## Methods and materials

### Study design, area and period

A community based cross sectional mixed methods study was conducted among 701 youths in Addis Ababa from September 1st to 30th, 2023. The current population of Addis Ababa is 5,461,000, with a growth rate of 4.46% [10]. Serving as the capital city of Ethiopia, Addis Ababa is presently divided into eleven sub-cities and 116 districts. These sub-cities include Addis Ketema, Akaki Kaliti, Arada, Bole, Kolfe Keranio, Lideta, Yeka, Kirkos, Nifas Silk, Gulele and Lume Kura. The residents of these sub-cities exhibit diversity in terms of ethnicity and socioeconomic status [11]. Addis Ababa is equipped with 12 public hospitals, 40 private hospitals, 96 health centers, and over 800 clinics. It comprises 12 Woredas, 722 blocks, 81,064 households, and 12 youth centers, each associated with specific Woredas. The study was conducted from the 1st to the 30th of September, 2023.

### Populations

Study population-all randomly selected out of school youths in the study area (quantitative) while the qualitative study involved all purposively selected out-of-school youths.

The source population for this study comprised all out-of-school youths residing in Addis Ababa. The study populations for this study were all randomly selected out of school youths in the study area (quantitative) while the qualitative study involved all purposively selected out-of-school youths. All out-of-school youths living in Addis Ababa were considered for inclusion in the study. However, individuals who were experiencing mental disabilities or facing serious illness during the data collection period were excluded. Additionally, out-of-school youths included in the quantitative study were not considered for participation in the qualitative study.

### Sample size determination and procedure

The sample size for this study was determined using the StatCalc program with the double population proportion formula, taking into account the following assumptions: a 95% level of confidence, 80% power, a proportion of outcome among the exposed (alcohol consumption) at 79.8%, a proportion of outcome among the non-exposed (no alcohol consumption) at 67.7%, an adjusted odds ratio of 1.1 [12], and accounting for a 10% non-response rate. This calculation yielded a sample size of 701 participants.

For the qualitative component, sixteen individuals participated in the focus group discussions, and 8 in-depth interviews were conducted in two groups. However, the qualitative research principle of saturation was adhered to, meaning the sampling continued until no new information or insights were obtained, ensuring thorough exploration of the topic [13].

The selection of study subjects followed a set of criteria, employing a multistage cluster sampling method. The primary sampling units were sub-cities, with four sub-cities randomly chosen from the eleven in Addis Ababa. The secondary sampling units were districts, randomly selected from the primary sampling units, with one district chosen from each sub-city. Subsequently, 30% of Ketenas, the smallest geographical units within districts, were randomly selected from the chosen districts. The sample size was then allocated to each selected Ketena based on household records from district offices, which contained information on the number and size of households.

In the randomly selected Ketenas, households were visited until the required number of youths for the interview was identified. Within each selected Ketena, participants were chosen randomly, and eligibility was determined using a short checklist. The initial household was selected by rolling a stick standing at the center of the Ketena and following a random direction. Subsequent households were systematically chosen every K^th^ until the required sample was identified.

For the qualitative part of the study, a purposive sampling technique was employed to select participants. Qualitative data were translated, transcribed, and coded using qualitative analysis. The information obtained from the qualitative component was then triangulated with quantitative findings as deemed appropriate.

### Variables

In this study, risky sexual behavior served as the dependent variable, while the independent variables were socio-demographic factors (age, educational level, income, and sex), personal factors (alcohol use, knowledge about HIV, and watching pornography), family factors (low-income Parental monitoring, and Parental communication) and friend factors (peer pressure and having friends who have high-risk sexual behavior).

### Operational definition

Out-of-School Youths: refers to youths aged 15–24 years, not attending day or night school or any vocational training, and unmarried at the time of the study [14].

Risk sexual behavior: refers to a participant will have either of the following: multiple sexual partners, early sexual start before the age of 18, sexual intercourse with commercial sex workers, unprotected sex (incorrect use of condom or flair to use the condom at least once during sexual intercourse [5].

Knowledge about HIV/AIDS: Participants who mentioned three or more transmission or prevention ways of HIV were categorized as having good knowledge; otherwise poor knowledge about HIV [15].

Multiple sexual partners: Those who had two or more lifetime sexual partners.

### Data collection tool and procedure

In the quantitative study, data were gathered through a structured questionnaire that was adapted and modified from sexual and reproductive health questionnaires developed by the World Health Organization and reviewed in various literature sources [5, 7, 8, 16]. The questionnaire encompassed sections on socio-demographic characteristics, personal factors, family-related factors, and questions related to sexual activity. Initially prepared in English, the questionnaire underwent translation into the local language, Amharic, by experts proficient in both languages. To ensure consistency, the translated version was then back-translated into English by different experts. The data were ultimately collected using the Amharic version of the questionnaire. In the qualitative study, guiding questions were formulated in the national language, Amharic. Transcripts were then translated back into English using a translator, facilitating the analysis and interpretation of qualitative findings.

Two separate questionnaires were employed for the study, with one designed for quantitative data collection and another for the qualitative component. The quantitative data were collected through face-to-face household interviews, which involved visiting households to gather the necessary information. In instances where the identified respondent was unavailable during the initial visit, arrangements were made for a follow-up appointment to conduct the interview. When multiple eligible participants were present in a selected household, one of them was randomly chosen to participate in the study. This approach ensured a systematic and unbiased selection process for study participants during the quantitative data collection phase.

For the qualitative aspect of the study, data were collected through focus group discussions (FGD) and in-depth interviews (IDIs). Both note-taking and audio recording methods were employed to comprehensively capture all the information shared during these sessions. The discussions and interviews were conducted in the local language, and the transcripts were later translated back into English to facilitate analysis and interpretation. To ensure a comfortable and open environment, female youths were moderated by a female moderator, while male youths were moderated by a male moderator. This approach likely contributed to a more conducive atmosphere for participants to express their perspectives and experiences during the qualitative data collection process.

### Data quality assurance

To uphold the quality of the study’s data, a series of meticulous steps were taken. Eight BSc health officers, fluent in Amharic, were enlisted as data collectors, and they underwent comprehensive two-day training on interviewing techniques, study objectives, and questionnaire sections. The questionnaire underwent a pre-testing phase involving 5% of the study population to assess its appropriateness, leading to necessary adjustments for improved clarity and relevance. Continuous supervision of the data collection process was carried out by both supervisors and the principal investigator. Daily scrutiny of the collected questionnaires ensured their consistency and completeness. These measures collectively served to enhance the accuracy and reliability of the data collected throughout the study.

In the qualitative segment, the transcripts for focus group discussions and in-depth interviews underwent a thorough process. Experienced and certified qualitative data transcribers and translators were engaged for this task. Two independent transcribers listened to the audio recordings and transcribed the respondents’ statements verbatim. Discrepancies between the audio records and transcribed text were meticulously verified through member checks, and any disparities were reconciled. Following this, the transcriptions were translated into English.

To uphold the trustworthiness of the study, key criteria such as credibility, dependability, confirmability, and transferability were carefully considered [17]. This comprehensive approach to transcription and translation aimed to ensure the accuracy, consistency, and reliability of the qualitative data, contributing to the overall credibility and validity of the study findings.

### Data processing and analysis

The collected data underwent a systematic process of coding, cleaning, and entry into EpiData version 3.1, subsequently being exported to SPSS version 25 for analysis. Descriptive statistics were employed to summarize the basic characteristics of participants, presenting proportions for categorical variables and mean with standard deviation (SD) or median with interquartile range (IQR) for continuous variables, depending on the data distribution.

Crude odds ratios (COR) with a 95% confidence interval (CI) were calculated to ascertain the crude associations between independent variables and high-risk sexual practices. Variables with a 𝑝-value ≤ 0.25 from the bivariate analysis were then selected for inclusion in the multivariable logistic regression model. The multivariable logistic regression analysis was conducted to control for confounding variables, with the model’s goodness of fit assessed using the Hosmer-Lemeshow statistic.

The strength of the association between dependent and independent variables was quantified using adjusted odds ratios (AOR) with a 95% confidence interval. Significance in statistical association was declared when the 𝑝-value was less than 0.05. This rigorous analytical approach aimed to unveil meaningful patterns and relationships within the data, providing robust insights into the factors influencing high-risk sexual practices among the study participants.

In the qualitative phase, the researcher meticulously handled the data, initiating the process by creating and organizing files that encompassed data collection, transcription, and translation to conduct phenomenological analysis. Subsequently, the translated data underwent a thorough reading and re-reading process until the complete meaning of the contents was comprehended. To enhance the validity and richness of the study, the information derived from the qualitative component was triangulated with quantitative findings where relevant. This methodological integration allowed for a comprehensive understanding of the research topic, enriching the overall analysis and interpretation of the study results.

## Results

### Socio demographic characteristics

This study included a total of 690 study participants, reflecting an impressive response rate of 98.4%. The age range of the participants varied from a minimum of 10 years to a maximum of 24 years, with a mean age of 17 ± 4 years. Notably, 234 individuals (33.9%) fell within the age bracket of 15–19 years. The study population comprised predominantly of females, with 57.2% participants, and a substantial proportion, 43.8% individuals, had no received formal education. Regarding religious practices, majority of the study participants (88%) attended daily. These demographic characteristics provide a comprehensive overview of the diverse composition of the study participants (Table 1).

**Table 1:** Socio demographic characteristics of out-of-school Youths in Addis Ababa, Ethiopia; 2023

Variables	Category	Percentage (%)
Age (in years)	10–14	33.5
	15–19	33.9
	20–24	32.6
Sex	Male	42.8
	Female	57.2
Educational status	No formal education	43.8
	Primary education	50.6
	Secondary education	5.7
Religion	Orthodox	73.5
	Muslim	20.9
	Protestant	5.2
	Others	0.4
Religious practice	Daily	6.7
	Once in a week	88.0
	Rarely	3.6
	Never	1.7
Fathers’ level of education	Unable to read and write	16.1
	Only read and write	24.8
	Primary	27.1
	Secondary	20.0
	College and above	12.0

### Personal characteristics

Among the study participants, a notable proportion, 8.6% individuals, reported having experience with watching pornography. Additionally, a substantial majority, comprising around three-quarters of the participants (74.6%), were found to be living together with both biological parents. Similarly, 20.3% participants reported living with either one of their biological parents, while the remaining 5.1% individuals resided with other family members or lived alone. Furthermore, approximately one-fifth of the study participants (20.9%) disclosed a habit of alcohol consumption. These findings provide insights into the prevalence of certain behaviors and living arrangements among the surveyed youth population. A study participant from an in depth interview was reported as:-

…Youths in the adolescence period are particularly vulnerable to HIV due to factors such as engaging in multiple sexual partnerships. The nature of adolescence often involves exploration and experimentation, which may lead to increased risk of unprotected intercourse without condom use. The absence of consistent one-to-one partnerships among many youths during this phase contributes to the heightened vulnerability to HIV transmission. It underscores the importance of targeted interventions and education efforts to address safe sexual practices and promote awareness of the risks associated with multiple sexual partners during this critical developmental stage.

### Family and related characteristics

The living arrangements of the study participants reveal that a significant majority, comprising three-fourths of them (74.6%), are residing with both of their biological parents. Another portion, 20.3% reported living with either one of their parents. Additionally, a substantial number of respondents, specifically 72.0% individuals, indicated having engaged in open discussions about sexual issues with their families. Furthermore, a considerable proportion of out-of-school youths, 78.8% individuals, reported being monitored by their parents. These findings shed light on the family dynamics and communication patterns related to sexual issues among the surveyed youth population (Table 2).

**Table 2:** Family and related characteristics of out-school youths in Addis Ababa, Ethiopia; 2023

Variables	Category	Percentage (%)
Living arrangement	Living with both biological parents	74.6
	Either of biological parents	20.3
	Others	5.1
Open discussion with families about sexual issue	Yes	72.0
	No	28.0
Parental monitoring	Yes	75.8
	No	24.2

### Peer and related characteristics

In the interviewed group of out-of-school youths, a substantial majority, 87.2% individuals, expressed engaging in open discussions with their friends about sexual issues. Additionally, more than half of the out-of-school youths, specifically 52.3% of individuals reported experiencing peer pressure. These findings highlight the prevalence of open communication among friends regarding sexual matters and the significant proportion of youths facing peer pressure within the studied population. A study participant from an FGD exploited as:-.

*“…Youths are particularly susceptible to the influence of their friends, often considering them as their closest companions. This strong bond can have a significant impact on their behavior, especially when it comes to adopting risky behaviors. If friends engage in high-risk activities, there’s a higher likelihood that the individual will be influenced to engage in similar behaviors. The peer environment plays a crucial role in shaping the choices and actions of youths, emphasizing the importance of fostering positive influences and providing support for healthy decision-making during this developmental stage”.*

### Prevalence of risky sexual behavior

Overall, the study found that 280 participants (40.6%) exhibited at least one of the risky sexual behavior practices. Thus, the prevalence of risky sexual behavior in this study was determined to be 40.6% (95%CI: 36.8%, 44.1%). Among them, a notable portion of the participants (9.6%%) reported experiencing unprotected sex, 18.8% of the study participants indicated initiating sexual activity before the age of 18, a smaller percentage (3.2%) of the study participants reported engaging in sexual activity with commercial sex workers, while 13.8% of the study participants disclosed having multiple sexual partners.

### Factors associated with risk sexual behavior

The odds of exhibiting risky sexual behavior among out-of-school youths in the age group 15–19 years old were found to be 2.52 times higher compared to those in the age group 10–14 years old, with an adjusted odds ratio (AOR: 2.52; 95%CI: 1.61 to 3.94). Similarly, female out-of-school youths were nearly three times more likely to engage in risky sexual behavior compared to their male counterparts (AOR: 2.84; 95% CI: 1.93 to 4.18). These findings highlight the age and gender differentials in the likelihood of participating in risky sexual behaviors among the out-of-school youth population. In contrast to this, a study participant from an in depth interview responded that:-.

*“…Youths, especially males in the adolescent period, often face exposure to environments like nightclubs, alcohol houses (mesheta bate), and peer pressure. This exposure can lead them into risky situations, making them vulnerable to exploited lifestyles, such as contact with commercial sex workers and involvement in street life. The confluence of these factors places them at a heightened risk of engaging in risky sexual behaviors. Understanding and addressing the unique challenges faced by youths in these environments is crucial for developing effective interventions to promote healthier choices and reduce the prevalence of risky behaviors among this vulnerable population”.*

Risky sexual behavior among out of school youths who have fathers who were unable to read and write was four times higher as compared with those who were studied college and above (AOR: 4.13; 95%CI: 2.04, 8.37). Similarly it was 2.45(AOR: 1.29, 4.63) and 2.13(1.10, 4.11) times higher among fathers who were studied primary and secondary education respectively.

Out of school youths who have a habit of alcohol consumption have 2.07 higher risk of having risky sexual behavior as compared with the counterparts who have no habit of alcohol consumption (AOR: 2.07; 95%CI: 1.33, 3.19). The odd of the likelihood of having sexual risk behavior among out of school children who have a peer pressure was 2.59 times higher as compared with those who have not peer pressure (AOR: 2.59; 95%CI: 1.81, 3.72). The study participants from FGD also confirmed that:*-*.

*“…Substance use, including alcohol, khat, and cigarette smoking, has been identified as a significant factor exposing school youths to various risky sexual behaviors, such as early sexual initiation, unprotected intercourse, and engagement with commercial sex workers. The influence of these substances may contribute to impaired judgment and decision-making, leading to increased vulnerability to such behaviors. Moreover, the pleasure derived from alcohol and khat use may contribute to a diminished perception of risk among youth regarding HIV/STIs. The altered state induced by these substances might impede individuals from adequately assessing the potential risks associated with their actions, thereby influencing their perception of susceptibility to health risks. Understanding the complex interplay between substance use and risky sexual behaviors is crucial for developing targeted interventions to address the specific challenges faced by youths and promoting healthier decision-making in these contexts”.*

Out of school youths who live together with one of biological parent have two folds higher risk of risky sexual behavior as compared with those who live with both biological parents (AOR: 2.32; 95%CI: 1.52, 3.55) (Table 3). A study participant from an in depth interview alleged that:-.

*“…Youths in the adolescence period face an increased vulnerability to risky sexual behavior due to several factors. Not being under the control of their families, lacking family support and affection, and experiencing exposure to peer pressure contribute to this heightened susceptibility. The combination of limited family guidance and the influence of peers during this critical developmental stage may lead to a greater likelihood of engaging in behaviors considered risky, such as unprotected sex or having multiple sexual partners. Understanding these dynamics is essential for designing effective interventions that address the unique challenges faced by youths and promote healthy decision-making in the realm of sexual behavior”.*

The odd of the likelihood of having risky sexual behavior among out of school youths who are watching pornography was two folds higher as compared with those who are not watching pornography (AOR: 2.10; 95%CI: 1.11, 3.97). Out of school youths who have parental monitoring were 41% less likely to have risky sexual behavior as compared with those who have no parental monitoring (AOR: 0.59; 95%CI: 0.39, 0.90). A study participant from an in depth interview expressed that:-

… the absence of family control, engaging in activities irresponsibly, a lack of appropriate income-generating activities, and being attracted by material goods, coupled with the inability to make informed decisions, collectively contribute to the vulnerability of youths. Similar to a newly released prisoner who gains newfound freedom, students may find themselves vulnerable to risky sexual behavior when they are free to make decisions outside the constraints of their family environment. This transition into more independence, coupled with the absence of proper guidance and financial stability, can create an environment where risky behaviors become more prevalent. Recognizing these factors is crucial for developing targeted interventions that support youths in making informed and responsible choices during this period of increased autonomy.

**Table 3:** Factors associated with risky sexual behavior among high-risk sexual practice among out-of-school Youths in Addis Ababa, Ethiopia; 2023

Variables	Category	RSB		COR(95%CI)	AOR(95%CI)
		Yes	No		
Age	10–14	79	152	1	1
	15–19	121	113	2.06(1.42, 2.99)*	2.52(1.61, 3.94)**
	20–24	80	145	1.06(0.72, 1.56)	1.13(0.73, 1.75)
Sex	Male	96	199	1	1
	Female	184	211	1.81(1.32, 2.47)*	2.84(1.93, 4.18)**
Fathers level of education	Unable to read and write	56	55	3.01(1.62, 5.58)*	4.13(2.04, 8.37)**
	Only read and write	63	108	1.72(0.96, 3.09)*	1.68(0.88, 3.21)
	Primary	77	110	2.07(1.16, 3.67)*	2.45(1.29, 4.63)**
	Secondary	63	75	2.48(1.36, 4.51)*	2.13(1.10, 4.11)**
	College and above	21	62	1	1
Open discussion	Yes	180	317	1	1
	No	100	93	1.89(1.35, 2.65)*	1.13(0.75, 1.69)
Alcohol consumption	No	200	346	1	1
	Yes	80	64	2.16(1.49, 3.14)*	2.07(1.33, 3.19)**
Peer pressure	Yes	185	176	2.59(1.89, 3.55)*	2.59(1.81, 3.72)**
	No	95	234	1	1
Living arrangement	With both biological parents	193	322	1	1
	One of biological parents	76	64	1.98(1.36, 2.89)*	2.32(1.52, 3.55)**
	Others	11	24	0.77(0.37, 1.59)	1.23(0.54, 2.78)
Watching pornography	Yes	30	29	1.58(0.93, 2.69)*	2.10(1.11, 3.97)**
	No	250	381	1	1
Parental monitoring	Yes	184	339	2.49(1.75, 3.55)*	0.59(0.39, 0.90)**
	No	96	71	1	1

*indicates variables having P-value < 0.25 in bivariate analysis and ** indicates variables having P-value < 0.05 in multivariable analysis

## Discussion

The prevalence of risky sexual behavior in this study was determined to be 40.6% (95% CI: 36.8, 44.1). This finding showed similarities with a study conducted in Kenya [2, 18], albeit slightly higher than the prevalence observed among school youth in Sri Lanka [19], Nigeria [20], and Ethiopia [21]. On the other hand, it was lower than the prevalence reported in studies among street children in Addis Ababa [22] and Dessie [18]. Several factors may contribute to this variation, including the age group difference among study participants, with this study encompassing a youth age range of 10–24 years compared to older age groups of 15–24 years in other studies. Consistently, socio-demographic and cultural variations between the study areas may account for the differences in observed prevalence rates. In addition, this also might be related to differences in exposure to sexual education (Out-of-school students may have limited access to comprehensive sexual education programs compared to their peers in formal educational settings. Lack of accurate information about sexual health and risk reduction strategies can contribute to higher rates of risky sexual behavior) [4, 7], peer influence and social works; where Out-of-school students may have different social networks and peer influences compared to in-school students. Peer pressure, social norms, and cultural influences within these networks can shape attitudes and behaviors related to sex and sexuality and Out-of-school students may face barriers to accessing sexual and reproductive health services, including contraception, STI testing, and counseling [11, 18, 21].

Age emerged as a significant factor associated with risky sexual behavior among out-of-school *youths* in this study. This finding aligns with similar conclusions drawn from studies conducted in Sri Lanka [23], Bahamas [24], Kinshasa [25], Gondar [26], Nekemte [27], and South Ethiopia [28]. The association between older age and increased likelihood of engaging in risky behavior might be attributed to factors such as higher drug use among older *youths*, contributing to their elevated risk of involvement in risky behaviors.

Sex was identified as a significant factor associated with risky sexual behavior among out-of-school youths, with female individuals demonstrating a nearly threefold higher risk compared to males. This finding is consistent with earlier studies conducted in Brazil [29] and Guduru, Ethiopia [30]. The possible explanation for this gender-based difference might be linked to the exchange of material goods within sexual relationships, including cash and cosmetics, and the occurrence of forced sex by males, whether within or outside the school setting. Moreover, the decision-making dynamics around condom use during sexual intercourse may contribute to this sex disparity, as male partners often make these decisions, potentially limiting females’ ability to advocate for safe sex compared to males. It’s noteworthy that the Shashamane study presented a reverse finding, indicating that males were 2.5 times more likely than females to engage in risky sexual behavior. This divergence may be suggestive of substance abuse, such as alcohol consumption and chat chewing, being more prevalent among males in Shashamane and contributing to risky sexual behavior [12].

Risky sexual behavior among out-of-school youths was found to be significantly associated with the educational level of their fathers. This finding underscores the influence of parental educational levels on the sexual behavior of out-of-school youths, highlighting the importance of family and parental factors in shaping *youths* behavior. Consistently, higher-educated fathers may provide greater financial stability and access to resources, which could potentially mitigate risky behaviors among their children [27]. Moreover, higher-educated fathers may prioritize education, health, and responsible decision-making, influencing their children to adopt similar values and behaviors [27, 28]. Conversely, lower-educated fathers may struggle to provide guidance on sexual health and risk reduction, leading to increased risky behavior among their children [26, 28].

Alcohol consumption was identified as a significant factor associated with risky sexual behavior among out-of-school youths. This finding is consistent with earlier studies conducted in Guduru, Ethiopia [30], Arba Minch [31], Bahir Dar [32], and Gondar [26]. The relationship between alcohol use and risky sexual behavior can be attributed to the decreased perception of risk associated with alcohol consumption. *Youths* under the influence of alcohol may experience a diminished sense of risk and engage in poor judgment, contributing to risky behaviors [33]. Additionally, studies in various contexts have highlighted that substance-using *youths*, including those consuming alcohol, are more likely to forego condom use and have a higher likelihood of unintended pregnancy [33, 34]. Moreover, the link between alcohol and risky sexual behavior is often explained by sensation-seeking behavior, characterized by a disposition to pursue novel and exciting levels of stimulation [34]. This disposition may contribute to *youths* engaging in risky behaviors, including those related to sexual activity, while under the influence of alcohol.

Peer pressure emerged as a significant factor associated with risky sexual behavior among out-of-school youths. This finding is consistent with studies conducted in Gondar [26], Guduru [30], Ethiopia, and Humera [35]. The influence of peer pressure on risky sexual behavior can be attributed to the tendency of *youths* to share their day-to-day life experiences with their friends, particularly among those living away from parents with poor parental monitoring. Y*ouths* often seek attention, recognition, and a sense of belonging from their peers, and this desire for social connection may influence them to adopt behaviors practiced by their intimate friends [36]. Furthermore, the tendency for individuals to form friendships with those who share similar attitudes and values may contribute to the observed link between peer pressure and risky sexual behavior [37]. The influence of peer pressure or motivation by friends can play a crucial role in shaping the behavior of *youths* in the realm of sexual activity.

Out-of-school youths who live together with only one of their biological parents were found to have a higher risk of engaging in risky sexual behavior. This association may be linked to the concept of family connectedness, which serves as a protective factor against risky sexual behavior. Numerous studies have demonstrated that positive relationships between parents and adolescents are associated with a reduced likelihood of using alcohol, tobacco, and drugs, as well as a lower likelihood of initiating sexual activity [38, 39]. The family environment, particularly the presence and support of both biological parents, can play a crucial role in shaping *youths behaviors* and providing a protective foundation against engaging in risky sexual activities.

Out-of-school youths who reported watching pornography were found to have a higher likelihood of engaging in risky sexual behavior. This finding aligns with a population-based study in Sweden, which indicated that boys who viewed pornography were more likely to participate in risky sexual practices [40]. Additionally, studies have suggested that individuals who frequently view or read pornographic materials are more likely to have multiple sexual partners compared to those who do not engage in such activities [41]. The link between watching pornography and risky sexual behavior may be attributed to the impulsive nature of pornographic materials, which can stimulate erotic thoughts and behaviors, potentially leading to engagement in risky sexual practices [36, 39]. Understanding the influence of media consumption on sexual behavior is essential for developing targeted interventions that address the potential impact of explicit content on *youths* attitudes and actions related to sexual activity.

Parental monitoring emerged as a significant protective factor associated with risky sexual behavior among out-of-school youths. This finding is consistent with numerous other studies and meta-analyses that have demonstrated the positive impact of family-focused interventions on delaying sexual intercourse and reducing risky sexual behavior among youths [38, 39, 41]. In contrast to some “youth-focused” prevention strategies that have shown limited effectiveness, interventions centered around enhancing parent-child communication, supportive parenting, and parental monitoring have consistently demonstrated positive effects on these outcomes [37]. The role of family dynamics, particularly the presence of supportive and monitoring behaviors by parents, plays a crucial role in mitigating the risk of engaging in risky sexual behavior among *youths* [36, 38].

Consistently, parents who actively monitor their children’s activities also serve as positive role models for responsible behavior [34, 35]. When adolescents observe their parents demonstrating healthy relationship dynamics, communication skills, and respect for boundaries, they are more likely to emulate these behaviors in their own relationships [36]. Parental monitoring not only influences adolescents’ behavior directly but also indirectly shapes their attitudes and beliefs about sex and relationships through observational learning [37, 38].

### Limitation of the study

While efforts were made to ensure acceptable quality assurance, it’s important to acknowledge that data on risky sexual behavior (RSB) were self-reported. This introduces a potential limitation, as the extent of under or over-reporting of behaviors cannot be precisely determined, and responses may be subject to social desirability bias. Additionally, it’s crucial to note that the study exclusively targeted out-of-school *youths*. As a result, the findings may not be fully representative of *youths* attending school in the same area. This limitation should be considered when attempting to generalize the study’s findings to the broader adolescent population, especially those in a school setting. The unique characteristics and circumstances of out-of-school *youths* may differ from those attending school, and caution should be exercised in applying the results to a more comprehensive context.

### Conclusion and recommendations

In this study, significant proportions of out of school youths have risky sexual behavior. Various factors independently associated with risky sexual behavior included age, sex, educational status of the husband, alcohol consumption, peer pressure, living arrangements with one biological parent, watching pornography, and family monitoring. Families should improve their communication with youths to reduce substance use and mitigate the impact of watching pornography. Families should support youths and strengthening the bond between them should be central to intervention efforts aimed at reducing risky sexual behavior among *youths*.

## Data Availability

No datasets were generated or analysed during the current study.

## Abbreviations

AOR: Adjusted Odds ratio
EDHS: Ethiopia Demographic Health Survey
HIV: Human Immune Virus
HRSP: High-Risk Sexual Practice
OSY: Out-of-School Youths
RSB: Risky sexual behavior
STI: Sexually Transmitted Infection
